# Number of Teeth and All-Cause and Cancer Mortality in a Japanese Community: The Takayama Study

**DOI:** 10.2188/jea.JE20180243

**Published:** 2020-05-05

**Authors:** Yuko Goto, Keiko Wada, Takahiro Uji, Sachi Koda, Fumi Mizuta, Michiyo Yamakawa, Chisato Nagata

**Affiliations:** Department of Epidemiology and Preventive Medicine, Gifu University Graduate School of Medicine, Gifu, Japan

**Keywords:** tooth loss, mortality, prospective studies

## Abstract

**Background:**

The association between the number of teeth and mortality among community-dwelling people has been examined in many epidemiological studies. However, few Japanese studies have included cancer mortality as an endpoint. We prospectively investigated the association between number of remaining teeth and all-cause and cancer mortality in a Japanese community.

**Methods:**

This study included participants in the Takayama Study who were aged 35–70 years old at baseline in 1992. Information on the number of remaining teeth was obtained from 11,273 participants via questionnaire at the second survey in 2002. The response rate was 66.9%. Deaths and their causes were ascertained during 11.8 years of follow-up.

**Results:**

A total of 1,098 deaths (435 cancer-related and 235 cardiovascular-related) were identified during the follow-up period. After adjusting for covariates, participants with 0 to 9 teeth were at moderate but significantly increased risk of all-cause mortality (hazard ratio [HR] 1.19; 95% confidence interval [CI], 1.03–1.39) and cancer mortality (HR 1.31; 95% CI, 1.03–1.67) compared to those with 20 or more teeth. With regard to cancer site, a significant association was observed for lung cancer (HR for 0–9 teeth vs. 20 or more teeth, 1.75; 95% CI, 1.08–2.83). This association was somewhat strengthened among never-smokers (HR 3.56; 95% CI, 1.02–12.45).

**Conclusions:**

We observed that a lower number of remaining teeth was significantly associated with increased risk from all-cause and lung cancer mortality. Further studies on the number of teeth and lung and other types of cancer are needed.

## INTRODUCTION

The association between the number of teeth and all-cause mortality has been examined in many epidemiological studies, as reviewed by Koka et al.^1^ However, many of these studies were based on narrowly selected populations, such as hospital patients. A review by Polzer in 2012 identified 15 prospective studies excluding those with such a select population.^2^ Overall, reduced number of teeth was associated with higher mortality, especially with cardiovascular disease (CVD)-associated mortality. Since that review, two additional studies of community-dwelling people have been published.^3^^,^^4^ Reduced number of teeth was associated with all-cause mortality and the risk of ischemic heart disease and peripheral vascular disease in the former smoker and alcohol consumption groups.

In Japan, to our knowledge, seven prospective studies have examined the association between the number of missing teeth/remaining teeth and all-cause mortality.^5^^–^^11^ All of them were conducted among community-dwelling people. Besides all-cause mortality, cancer mortality was included as an endpoint in only three studies,^6^^,^^7^^,^^9^ and one of them reported a significant association between the number of missing teeth and the risk of mortality from orodigestive cancer.^7^ In that study, an oral examination was conducted at baseline. However, the number of study subjects was small (*n* = 656), and the subjects were restricted to elderly people (mean age, 80 years old). Tooth count based on oral examination has an advantage over that based on self-report because misclassification is less likely to occur. However, it would be difficult to conduct an oral examination in sufficiently large samples that can be used for cancer mortality analysis. In fact, two other studies based on oral examination included only all-cause mortality as an endpoint.^5^^,^^10^ On the other hand, the self-reported number of teeth has been reported to have good correlation with the actual number of teeth based on oral examination in a general population.^12^ We aimed to prospectively examine the association between the number of teeth and all-cause and cause-specific (cancer, CVD, and other-cause) mortality in Japanese men and women aged 45 years or older living in a community in Japan. Although the number of teeth was self-reported in our study, the sample size was larger than in any previous study in Japan.

## MATERIALS AND METHODS

This study was a follow-up study of participants in the Takayama Study. The Takayama Study was initiated in 1992, and 31,552 residents in Takayama City aged 35 years or older responded to a questionnaire on demographic characteristics, smoking and alcohol habits, diet, physical exercise, and medical and reproductive histories. The response rate was 85.3%. The study design and methodology of the Takayama Study have been previously described.^13^ The second survey was conducted in July 2002 and restricted subjects to those who were younger than 70 years old at the first survey. After excluding those who were known to be dead, to have relocated, or to be physically unable to complete the questionnaire, 22,394 individuals were invited to the second survey, and 14,971 (66.9%) responded to the self-administered questionnaire. The questionnaire asked, “How many natural teeth do you have remaining?”. We did not request participants to count the number of teeth excluding third molars. The questionnaire also asked about height and weight, smoking status, marital status, physical activity, and medical and reproductive histories. We excluded participants who reported a medical history of coronary heart disease (*n* = 947), stroke (*n* = 203), or cancer (*n* = 628) and those with unknown data about the number of teeth (*n* = 1,920). Thus, 11,273 (4,968 men and 6,305 women) participants were included in the present analyses. This study was approved by ethics board of the Gifu University Graduate School of Medicine.

All deaths and their causes during the follow-up period from 2002 to 2013 were identified using death certificates provided by the Legal Affairs Bureau, Japan. The causes of death were coded according to the International Classification of Disease (ICD), 10th Revision (ICD-10). The endpoints of this study were deaths from all causes, as well as death from cancer (C00–D48), cardiovascular disease (I00–I99), and all other causes. To explore the association between number of teeth and cancer mortality, mortality from upper gastrointestinal cancer, including esophageal (C15) and stomach (C16) cancers; that from orodigestive cancers, such as cancer in the lip, oral cavity, and pharynx (C00–C14), esophagus (C15), stomach (C16), pancreas (C25), liver (C22), and colon, rectum, and anus (C18–C21); and that from all cancers other than lung cancer. An increased risk of these cancers associated with lower number of teeth has been reported in some previous studies.^7^^,^^14^ Furthermore, chronic obstructive pulmonary disease (COPD)-related causes, including obstructive chronic bronchitis, emphysema, chronic obstructive asthma, and bronchiectasis (J41–J47), were included as endpoints. Orodigestive cancer^7^ and COPD-related causes^15^ were defined previously. Information on participants who had moved away from Takayama was obtained from residential registers or family registers. During the study period, 277 individuals moved out of the study area, and the date of emigration was unknown for 50 of them. They were recorded in a census at the latest date when they were known to reside in the city.

A follow-up period was established for each participant from the second survey (July 1, 2002) to the date of death, the date of emigration out of Takayama, or end of the study (March 31, 2013), whichever occurred first. Subjects were divided into three groups according to the number of teeth: 20 or more, 10–19, and 0–9. We used the Cox proportional hazards model to estimate the hazard ratio (HR) and 95% confidence interval (CI) for all-cause and cause-specific mortality for each category as compared to the category for the highest number of teeth. The number of teeth (continuous variable) was used to assess the linear trend. Age, sex, education level (≤11, 12–14, or ≥15 years), body mass index (BMI) in quartile, physical activity score (METs-h/week), alcohol consumption in quartile, smoking status (never, former with <30 pack-years of smoking, former with ≥30 pack-years of smoking, current with <30 pack-years of smoking, or current with ≥30 pack-years of smoking), marital status (married or not married), and medical history of diabetes and hypertension (yes or no) were included in the model as covariates. For information on alcohol consumption and education level, data from the first survey were used because we did not obtain those data in the second survey. As a sensitivity analysis, we conducted competing risk regression analysis using method proposed by Fine and Gray.^16^ The association of the number of teeth with cancer mortality was assessed with non-cancer death as a competing event. For the association with lung cancer mortality, death from other causes was a competing event. Subgroup analysis according smoking status (never, current or former with <30 pack-years of smoking, and current or former with ≥30 pack-years of smoking) were conducted. To test the hypothesis that there was no association modification between each covariate and the number of teeth with regard to the risk of mortality, we used the likelihood ratio test to compare the model that included the different combinations of the number of teeth and the potential effect modifier with a model that included number of teeth and the potential effect modifier as separate variables. All *P*-values were calculated using a two-sided test, and a *P*-value of less than 0.05 was considered statistically significant in all analyses. All statistical analyses were performed using the SAS program (SAS Institute, Cary, NC, USA).

## RESULTS

Age of study participants ranged from 45 to 79 years. The mean of number of remaining teeth was 18.0 (standard deviation, 9.9). Table 1 shows basic characteristics according to the number of remaining teeth at the baseline. Participants who had more teeth were more likely to be young, married, more educated, less obese, physically active, and never-smokers and less likely to have reported a history of hypertension and diabetes mellitus. During the follow-up (median, 10.2 years), 1,098 participants died (711 men and 387 women).

**Table 1.** Basic characteristics according to number of teeth

Variable	Number of teeth			

	≥20	10–19	0–9	*P*-value
Sex, *n* (%)	6,174 (54.8)	2,478 (22.0)	2,621 (23.2)	0.01
Male	2,765 (44.8)	1,028 (41.5)	1,175 (44.8)	
Age, years	57.6 (8.1)	62.4 (8.2)	67.4 (7.4)	<0.001
Married, *n* (%)	5,431 (88.5)	2,053(83.6)	2,068 (79.5)	<0.001
Years of education, *n* (%)				<0.001
≤11	2,643 (43.0)	1,485 (60.2)	1,894 (73.2)	
12–14	2,805 (45.7)	837 (34.0)	589 (22.8)	
≥15	693 (11.3)	143 (5.8)	105 (4.0)	
BMI, kg/m^2^	22.5 (2.8)	22.4 (2.8)	22.2 (3.0)	<0.001
Physical activity, METs-h/week	35.6 (47.2)	30.2 (45.0)	26.5 (39.3)	<0.001
Alcohol consumption, g/day	24.0 (34.2)	22.3 (32.3)	22.0 (34.6)	<0.001
Pack-years of smoking, *n* (%)				<0.001
0	3,581 (58.3)	1,426 (58.1)	1,425 (55.0)	
Past,<30 pack-years	596 (9.7)	200 (8.2)	239 (9.2)	
Past, ≥30 pack-years	774 (12.6)	383 (15.6)	404 (15.6)	
Current, <30 pack-years	876 (14.3)	272 (11.1)	275 (10.6)	
Current, ≥30 pack-years	314 (5.1)	173 (7.0)	250 (9.6)	
History of hypertension, *n* (%)	1,289 (20.9)	641 (25.9)	774 (29.5)	<0.001
History of diabetes mellitus, *n* (%)	322 (5.2)	161 (6.5)	215 (8.2)	<0.001

BMI, body mass index; MET, metabolic equivalent.

All values are given as means (standard deviation) or percentages.

*P*-values were based on linear regression analysis for continuous variables and the Chi-square test for categorical variables.

Table 2 shows the age- and sex-adjusted and multivariate-adjusted hazard ratios (HRs) of all-cause and cause-specific mortality according to number of teeth. As there was no significant interaction by age, sex, and others covariates, they were included in the model as potential confounders. For example, the *P*-value for interaction terms by sex for all-cause mortality was 0.65.

**Table 2.** Hazard ratios of all-cause and cause-specific mortality according to number of teeth in the Takayama study

Variable	Number of teeth				

	≥20	10–19	0–9	Per 1 tooth lost	*P*-value^a^
**All causes**					
Number of death	367/6,174	274/2,478	457/2,621		
Age and sex adjusted^b^	1	1.25 (1.07–1.47)	1.31 (1.13–1.53)	1.01 (1.01–1.02)	<0.001
Multivariate adjusted^c^	1	1.18 (1.01–1.39)	1.19 (1.03–1.39)	1.01 (1.00–1.01)	0.01
**All cancer**					
Number of death	155/6,174	105/2,478	175/2,621		
Age and sex adjusted^b^	1	1.27 (0.98–1.63)	1.45 (1.15–1.84)	1.02 (1.01–1.03)	0.001
Multivariate adjusted^c^	1	1.18 (0.92–1.53)	1.31 (1.03–1.67)	1.01 (1.00–1.02)	0.02
**Lung cancer**					
Number of death	32/6,174	27/2,478	54/2,621		
Age and sex adjusted^b^	1	1.61 (0.95–2.71)	2.19 (1.36–3.54)	1.03 (1.02–1.05)	0.001
Multivariate adjusted^c^	1	1.39 (0.82–2.34)	1.75 (1.08–2.83)	1.02 (1.00–1.04)	0.03
**Upper gastrointestinal cancer**					
Number of death	31/6,174	25/2,478	22/2,621		
Age and sex adjusted^b^	1	1.51 (0.90–2.66)	0.95 (0.52–1.71)	0.99 (0.97–1.02)	0.70
Multivariate adjusted^c^	1	1.50 (0.87–2.60)	0.88 (0.48–1.61)	0.99 (0.97–1.02)	0.50
**Orodigestive cancer**^d^					
Number of death	78/6,174	54/2,478	77/2,621		
Age and sex adjusted^b^	1	1.27 (0.89–1.82)	1.25 (0.88–1.76)	1.01 (0.99–1.02)	0.15
Multivariate adjusted^c^	1	1.22 (0.85–1.74)	1.16 (0.82–1.65)	1.01 (0.99–1.02)	0.33
**All cancer other than lung cancer**					
Number of death	129/6,174	78/2,478	121/2,621		
Age and sex adjusted^b^	1	1.18 (0.88–1.58)	1.26 (0.96–1.66)	1.01 (0.99–1.02)	0.06
Multivariate adjusted^c^	1	1.13 (0.84–1.51)	1.19 (0.90–1.58)	1.01 (0.99–1.02)	0.17
**CVD**					
Number of death	78/6,174	65/2,478	92/2,621		
Age and sex adjusted^b^	1	1.22 (0.87–1.70)	0.98 (0.71–1.36)	1.00 (0.99–1.01)	0.99
Multivariate adjusted^c^	1	1.18 (0.84–1.65)	0.92 (0.67–1.28)	1.00 (0.98–1.01)	0.70
**Other causes**					
Number of death	129/6,174	97/2,478	185/2,621		
Age and sex adjusted^b^	1	1.23 (0.94–1.61)	1.42 (1.11–1.81)	1.02 (1.01–1.03)	0.002
Multivariate adjusted^c^	1	1.17 (0.89–1.53)	1.27 (0.99–1.62)	1.01 (1.00–1.02)	0.04
**COPD-related**					
Number of death	3/6,174	5/2,478	15/2,621		
Age and sex adjusted^b^	1	2.37 (0.56–10.03)	3.74 (1.04–13.50)	1.05 (1.00–1.11)	0.04
Multivariate adjusted^c^	1	1.90 (0.44–8.20)	2.68 (0.73–9.80)	1.04 (0.99–1.10)	0.10

COPD, chronic obstructive pulmonary disease; CVD, cardiovascular disease.

^a^*P*-value for the risk per 1 tooth lost.

^b^Adjusted for age and sex.

^c^Adjusted for age, sex, body mass index, pack-years of smoking, alcohol consumption, education level, marital status, physical exercise, and medical history of hypertension and diabetes mellitus.

^d^Orodigestive cancer included as cancers in lip, oral cavity and pharynx, esophagus, stomach, pancreas, liver and colon, rectum, and anus.

Participants with 0 to 9 teeth were at moderately but significantly increased risk of all-cause mortality as compared to those with 20 or more teeth after controlling for the covariates (HR 1.19; 95% CI, 1.03–1.39). The trend was also statistically significant (*P* = 0.01). Participants with 0 to 9 teeth were also at increased risk of all-cancer mortality (HR 1.31; 95% CI, 1.03–1.67) but not of cardiovascular mortality. Among cancer sites, a significant risk increase was observed for mortality from lung cancer (HR for 0–9 teeth vs. 20 or more teeth, 1.75; 95% CI 1.08–2.83, *P* for trend = 0.03). There were no significant associations between the number of teeth and mortality from upper gastrointestinal cancer, orodigestive cancer, and all cancers other than lung cancer. Exclusion of deaths during the first 3 years did not alter the results substantially; the HRs of all-cause and lung cancer mortality for the lowest versus the highest number of teeth categories were 1.18 (95% CI 1.01–1.40, *P*-trend = 0.05) and 1.98 (95% CI 1.18–3.33, *P*-trend = 0.02), respectively.

A competing risk analysis demonstrated a similar pattern of results: the HRs of all cancer and lung cancer mortality for the lowest versus highest number of teeth categories were 1.37 (95% CI 1.02–1.68, *P*-trend = 0.03) and 1.74 (95% CI 1.05–2.89, *P*-trend = 0.04), respectively.

The association between number of teeth and lung cancer mortality was somewhat stronger among never-smokers (Table 3). The associations did not differ greatly according to smoking status and smoking intensity level.

**Table 3.** Associations between smoking status and lung cancer mortality

Variable	Number of teeth			

	≥20	10–19	0–9	*P*-value
**Never smoker**				
Number of death	5/3,581	3/1,426	10/1,425	
Multivariate adjusted^a^	1	1.25 (0.29–5.44)	3.56 (1.02–12.45)	0.05
**Current and past smokers with <30 pack-years**				
Number of death	7/1,472	8/472	18/514	
Multivariate adjusted^a^	1	1.99 (0.71–5.61)	2.52 (0.99–6.42)	0.06
**Current and past smokers with ≥30 pack-years**				
Number of death	20/1,088	16/556	26/654	
Multivariate adjusted^a^	1	1.25 (0.64–2.45)	1.33 (0.69–2.53)	0.45

^a^Adjusted for age, sex, body mass index, alcohol consumption, education level, marital status, physical exercise, and medical history of hypertension and diabetes mellitus.

## DISCUSSION

This study found that a lower number of remaining teeth was significantly associated with the risk of all-cause as well as cancer mortality. Increased risk of cancer mortality appeared to be due to the increased risk of mortality from lung cancer. Our findings for lung cancer are consistent with some previous studies.^17^^–^^19^ So far, to our knowledge, four prospective studies (five publications),^7^^,^^14^^,^^17^^,^^20^ including one among Japanese elderly people, examined the association between number of teeth and risk of lung cancer or risk of mortality from lung cancer. Only one study, the Health Professionals Follow-Up Study, observed a significant higher risk of lung cancer with a lower number of remaining teeth after adjustment for smoking.^19^ One case-control study among Japanese adults reported a positive association after controlling for smoking status, but the association was obvious among current smokers.^18^ Shi et al, in a meta-analysis of two prospective studies, showed that for every 10 teeth lost, there was a 19% increase in lung cancer risk.^21^ There has been concern that the association between number of teeth and lung cancer, as well as other smoking-related diseases such as cardiovascular disease, may be due to the residual confounding effect of smoking. In fact, in the Health Professionals Follow-Up Study, exclusion of smokers attenuated the association between the number of teeth and the risk of lung cancer.^19^ However, in our study, the association between number of teeth and lung cancer mortality was not attenuated among never-smokers, and the associations did not differ by smoking intensity level, suggesting that smoking is not likely to account for the excess risk of mortality from lung cancer.

The mechanism behind the link between number of teeth and lung cancer is unclear. Tooth loss is likely to be the results of untreated or unsuccessfully treated periodontal disease and/or caries.^22^^,^^23^ Chronic infection and inflammation due to periodontitis or caries may have implications in the pathogenesis of cancer. However, we did not observe a significant association of number of teeth with mortality from other types of cancer. As Hujoel et al suggested,^17^ the aspiration of dental biofilm may cause lung colonization, which may cause obstructive pulmonary disease and, in turn, lead to lung cancer. In fact, we observed that the fewer number of teeth was positively associated with the risk of mortality from COPD-related disease, although the association was not significant due to the small number of cases. The potential link between tooth loss without periodontal disease or caries and the lung cancer mortality should be considered. Tooth loss has been associated with altered diet and reduced intake of most nutrients^24^^,^^25^ because of insufficient mastication. Malnutrition due to tooth loss may have impact on the prognosis rather than the incidence of lung cancer. Such impact may be more manifested in cancers with poor prognosis, like lung cancer. Although some cancers, such as pancreatic and esophageal cancers, are also known to have poor prognosis, the sample size of the present study was insufficient to conduct analyses for individual type of cancer with small number of deaths. We observed a somewhat strong association between the number of teeth and lung cancer mortality among never smokers. Such association may have been more likely to be revealed among never smokers, as the major determinant of the incidence as well as the prognosis of lung cancer, smoking,^26^ was absent among them. Exclusion of deaths during the first 3 years did not alter the results. However, it is possible that underlying illness or preclinical symptoms due to lung cancer may have caused tooth loss.

The strengths of our study include the prospective design, representation of the community-dwelling population, the relatively large sample size, the high response rate and long length of follow-up, information on potential confounders, and restriction to never-smokers. There were also several limitations. Although the self-reported number of teeth has been reported to be correlated with the actual number of teeth,^12^ the use of a self-reported questionnaire is a major limitation of our study. It is possible that some subjects reported the number of missing teeth but not remaining teeth. However, such misclassification should be unlikely to depend on death or lung cancer death and could have resulted in attenuation of the associations. Although we considered the potential confounding and modifying effect of smoking, which is critical for the association between number of teeth and smoking-related diseases, we cannot deny confounding due to the unmeasured aspect of smoking history or unknown factors. Furthermore, we could not obtain information on denture use. Although denture use has been suggested to have a beneficial effect with regard to mortality in some studies,^27^^,^^28^ the results are not conclusive.^2^ In addition, Murai et al reported that the usage rate of removable partial dentures increased as the number of missing distal extension teeth and bilaterally missing teeth increased.^29^ It is unlikely that the observed inverse association between the number of teeth and all-cause and lung cancer mortality is due to increased use of dentures among those with a higher number of remaining teeth. The sample size was limited, which precluded analyses of various types of cancer with small numbers of deaths.

In summary, we observed that a fewer number of remaining teeth was significantly associated with an increased risk of all-cause and lung cancer mortality. These associations were not attenuated among never-smokers. Although the present study suggested a possible link between number of teeth and lung cancer mortality, studies on the number of teeth and lung cancer are scarce, and the results have been inconsistent. In addition, the underlying mechanism is unclear, and we cannot deny the possibility of associations with not only lung but also other types of cancer. Therefore, further studies on the number of teeth and lung and other types of cancer are needed.
